# Deviation of peak hours for metro stations based on least square support vector machine

**DOI:** 10.1371/journal.pone.0291497

**Published:** 2023-09-13

**Authors:** Lijie Yu, Mengying Cui, Shian Dai

**Affiliations:** 1 College of Transportation Engineering, Chang’an University, Xi’an, Shaanxi, China; 2 Department of Civil and Architectural Engineering, Xi’an Jiaotong University City College, Xi’an, Shaanxi Province, China; Shenzhen University, CHINA

## Abstract

The station-level ridership during the peak hour is one of the key indicators for the design of station size and relevant facilities. However, with the operation of metro system, it cannot be ignored that, in many cities, the station peak and the city peak may not be simultaneously occurred. As the current ridership forecasting methods use the city peak as the point of reference, stations with wide differences of ridership in between would experience disorders due to serious underestimates of passenger demand during the actual peak. Accordingly, this study fully considers the phenomenon that the metro station peak is not identical to the city peak and focuses on the concept of the peak deviation coefficient (PDC), the ratio of the station peak ridership to the city peak ridership. It investigates how metro ridership determinants affects the PDC using the least square support vector machine (LSSVM) model. A land-use function complementarity index is employed as one of the independent variables, which is newly proposed in this study that describes the relationship of the commute land use around an individual station with that along the whole network. This method can help to resolve the ridership amplification indicator for a fine-grained station-level forecasting. The results for Xi’an metro indicate that the LSSVM is an effective method to scrutinize the nonlinear effects of e.g., land use attributes, on the temporal distribution features of the metro ridership. Compared to the ratio of commute land use measured for individual stations, the land-use function complementarity index can better explain and predict the severity of peak deviation phenomenon, controlling other independent variables in the model.

## Introduction

Given the long service cycle of the metro system, accurate predictions of metro ridership are necessary when designing the lines and building the stations for future transit demand. It has been recognized that the station-level ridership varies during the time of day [1,2]. Among all the time slots, the peak hour ridership is one of the keys for the capacity design of the station service facilities as well as the formulation of the operation organization scheme. In the previous studies, many scholars consider the peak as a fixed period of time based on the average hourly ridership overall, e.g., the morning (7:00–8:00 a.m.) or evening peak (6:00–7:00 p.m.) [3,4], hereafter called as the city peak.

However, with the increase of land use intensity and diversity, especially along the metro lines, a phenomenon shows up that the maximum hourly ridership of a station in a day, named as the station peak, is not exactly occurred during the city peak. In other words, metro stations may have a distinct peak period of passenger flow, compared to the city peak We define such a phenomenon as the peak deviation for metro stations. Gu and Ye [5] scrutinized the urban rail transit data in both Shanghai and Osaka and verified the existence of the peak deviation. Yu et al. [6] focused on the Xi’an metro and found that about 65.2% of metro stations have their station peaks shift from the city peak. Similar findings also remain for the case of Beijing [7] and Nanjing [8], China.

Traditional macroscopic forecasting methods, such as the four-step model, is widely applied for metro passenger flow forecasting in real-world implementations nowadays, which, however, set the city peak as the peak time parameter and ignore the peak deviation for metro stations in particular [9,10]. The stations designed accordingly would experience disorders to some extent, given the fact that, if the peak deviation exists, their actual peak flow can be greater than the predicted city peak flow, so the size of the stations and the required supporting equipment are underestimated [11]. Considering this practical issue, in the year of 2016, the Chinese government updated the national standard for the *Code of Metro Ridership Forecast*, and claimed that a separate peak hour ridership forecast should be conducted for stations that show the deviation of peak hours.

There were studies pointed out that the location of metro stations as well as the built environment nearby should determine the peak deviation [5,12]. However, until now, few researchers have explored what exactly affect it and how the effects are. Yu et al. [6] used four different clustering methods to categorize the metro station into five groups based on selected peak deviation indexes, e.g., ridership changes between the city and station peak, and qualitatively compared the features of surrounding land use attributes for each. Yu et al. [12] further examined the spatial impacts of land use factors on the peak hour ridership using a geographically weighted regression model.

Hence, in this study, we fully consider the phenomenon that the metro station peak is not identical to the city peak and focus on the concept of the peak deviation coefficient (PDC), the ratio of the station peak ridership to the city peak ridership, defined by Yu et al. [6]. The objective of this study is to explore the influencing factors of the PDC using the Least Square Support Vector Machine (LSSVM), from a quantitative analysis perspective, which helps to determine the ridership amplification indicator for a fine-grained station-level passenger flow forecasting. The findings would provide critical evidence for planners to balance the time utilization of the metro system.

Note that a land-use function complementarity index is employed as an independent variable in the model to explain the interaction strength between stations on the network [13]. Recent studies in transit direct ridership models highlighted the competitive and complementary inter-station relationship [14–16]. We believe such a mutual impact also affect the peak deviation of a single station.

The rest of the paper is organized as follows. Section Concept of PDC introduces the generation mechanism of the peak deviation for metro stations and clarifies the definition of the PDC. Section Method illustrates the methodology, including the specifications of selected independent variables and also an interpretation of the LSSVM method. 2021 Xi’an metro is then selected as the case study. The results of the PDCs for both boardings and alightings in the morning and evening are elaborated in Section Case study Section Conclusion concludes the study and puts forward some future works.

## Concept of PDC

The city peak expresses a time period of the day during which passenger flow on the whole metro network is at its highest. Assume that the city peak emerges at time slot *t*^*c*^, the passenger flow during then can be written as,

$$P_{i,t^{c}}=\overset{t^{c}+\Delta t}{\underset{t^{c}}{\int }}P_{i}(t)dt$$ (1)

where:

$P_{i,t^{c}}$ is the station-level passenger flow in station *i* during the city peak,

*P*_*i*_(*t*) is the passenger flow in station *i* at time slot *t*,

*t*^*c*^ is the start time of the city peak,

Δ*t* is the time interval counting the peak period, and normally it is set as 1 hour, e.g., 8am - 9am or 6pm - 7pm.

While the station peak concentrates on a single station in separate, which refers to a part of the day that the station has its maximum ridership.

$$P_{i}(t_{x})=\overset{t_{x}+\Delta t}{\underset{t_{x}}{\int }}P_{i}(t)dt,\;(0\leq x\leq 24)$$ (2)

$$P_{i,t_{i}^{s}}=\max_{x}(P_{i}(t_{x}))$$ (3)

where:

$P_{i,t_{x}}$ is the passenger flow in station *i* between the time slot *t*_*x*_ and *t*_*x*_+Δ*t*,

$P_{i,t_{i}^{s}}$ is the passenger flow in station *i* during the station peak starting from time slot $t_{i}^{s}$.

Accordingly, two points need to be accentuated,

- For a station in the network, *t*^*c*^ is not necessarily equal to $t_{i}^{s}$, which means that the station peak of a station may not be simultaneously occurred with the city peak. To be more specific, two scenarios could arise,
- When $t^{c}=t_{i}^{s}$, no peak deviation can be observed, so $P_{i,t^{c}}=P_{i,t_{i}^{s}}$ are the same;
- When $t^{c}\neq t_{i}^{s}$, the deviation between the city peak and the station peak can be observed, so $P_{i,t^{c}}<P_{i,t_{i}^{s}}$.
- $t_{i}^{s}$ is not necessarily equal to $t_{j}^{s}$, as different stations may not share the same station peak.

The cause of the peak deviation for metro stations can be explained considering the mechanism of trip generation.

Specifically, a station, as the link to connect the metro system to the external environment, gathers and distributes individual trips produced or attracted by different land uses around itself. Given that individual travel behavior can be random in one sense, but the aggregation at different times of the day has potential regularity, the temporal distribution of passenger flow in a station should be equal to the weighted sum of the temporal distribution of individual trips regarding different land uses.

$$P_{i}(t)=\underset{n}{\sum }\beta_{i,n}q_{n}(t)$$ (4)

where:

*P*_*i*_(*t*) is the passenger flow at time slot *t* in station *i*,

*q*_*n*_(*t*) is the number of individual travels produced or attracted by land-use *n* at time slot *t*,

*β*_*i*,*n*_ is the proportion of type *n* travels in station *i*.

The value of *β*_*i*,*n*_ in station *i* is distinct from other stations because of the different built environment surrounded, thus generates disparate *P*_*i*_(*t*). As a result, the start time of the station peak $t_{i}^{s}$ can be affected by *β*_*i*,*n*_, indicating that the type of dominant land use around a station can largely determine the station peak.

**Fig 1:**
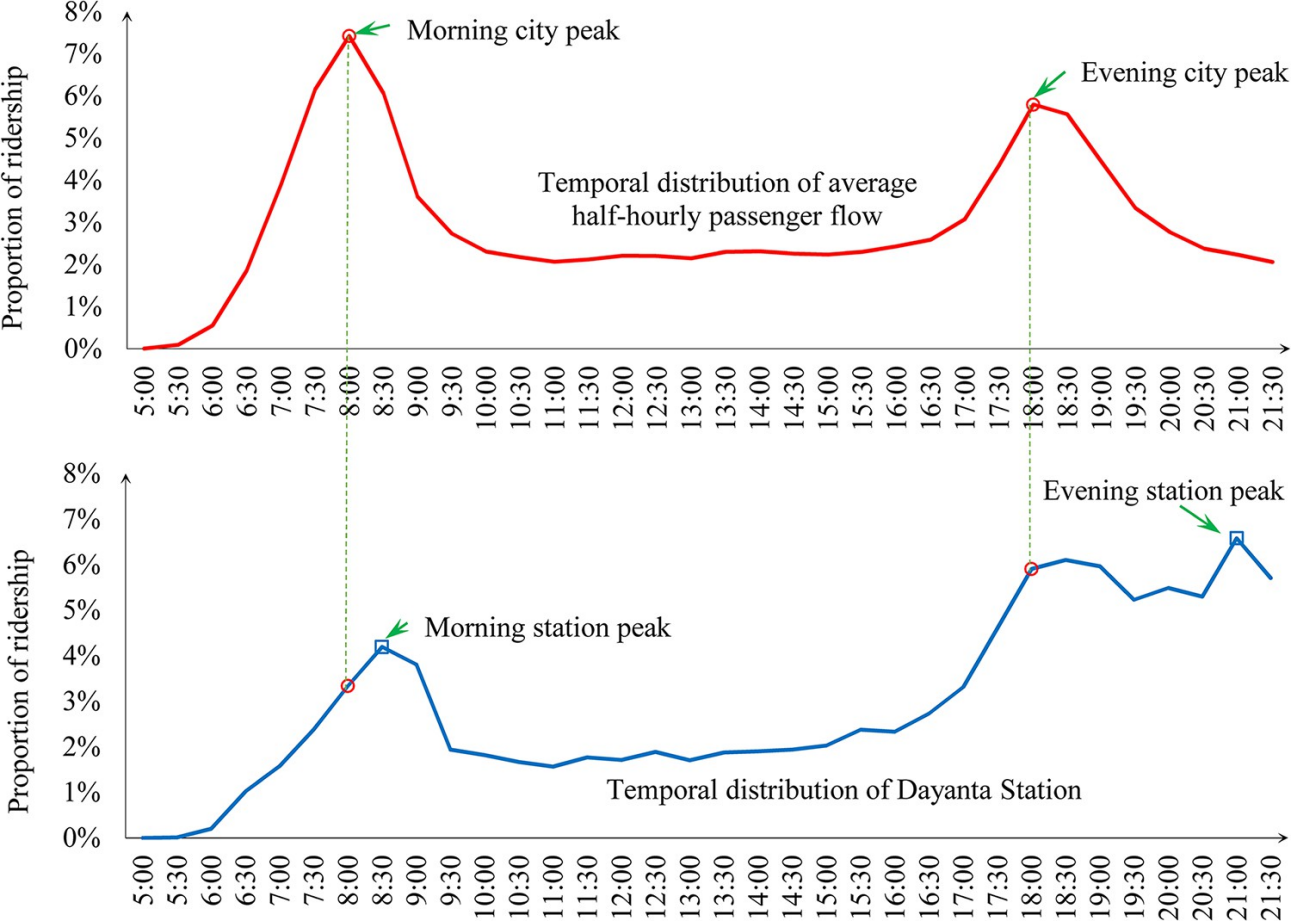
Temporal distribution of average half-hourly passenger flow and the metro Dayanta station.

Fig 1 illustrates the difference between the city peak and the station peak by displaying the temporal distribution of the average half-hourly passenger flow on the metro network overall as well as that of Dayanta Station in Xi’an Metro (data in weekdays are selected for this comparison). It is clearly that two city peaks can be recognized, 7:30–8:30 a.m. and 18:00–19:00 p.m. respectively, manifesting the dominance of commuting travel on weekdays. Dayanta station, close to a celebrated scenic spot—Greater Wild Goose Pagoda, has its station peak shift to 8:30–9:30 a.m. in the morning and 21:00–22:00 p.m. in the evening, as leisure tourism travels are dominated.

To improve the prediction accuracy of the maximum ridership at the station-level, it is necessary to identify the station peak. Because the station peak may not be appeared at the same time, and the macroscopic forecasting model has better control effect on the total error, this paper direct to put forward an amplification factor, which can be used to amend the predicted volume from the city peak hour to the station peak hour. It is written as,

$$PDC_{i}=\frac{P_{i,t_{i}^{s}}}{P_{i,t^{c}}}$$ (5)

where:

*PDC*_*i*_ is the peak deviation coefficient of station *i*.

From Eq 5, we can see that the value of PDC is no less than 1.

## Method

### Independent variables

Passenger satisfaction towards the subway system can be affected by the size of the stations from the perspective of comfort [17] as well as efficiency [18], so the prediction of the passenger flows in and out of a station is the fundamental for the organization and management of the urban rail transit system. Factors determining the ridership at the station-level include many categories, e.g., land use attributes and built environments [9], population or jobs, access features [19], socioeconomic factors [20], passenger travel characteristics [21], and features of stations [22].

Research on the peak deviation for the subway stations is still in the primary stage. We can see it as a type of studies looking at the station-level ridership prediction, but spotlight the temporal distributions. Therefore, this paper considers to use the determinants of metro ridership as the independent variables when explaining the PDC. Details are discussed as follows.

#### Commute land use

Many studies have found that land use is closely related to the metro ridership [23,24]. Undoubtedly, as a parameter that affects the temporal distribution of passenger flows, the PDC is also affected by the surrounding land use attributes. In general, resident trips can be divided into two categories, namely commute and non-commute travels. Qualitatively, a larger percentage of recreational land use near a station would lead to fewer commuting trips, so its PDC might be higher. Thus, the features of land use that induces the commute travels, hereafter called as commute land use, should be able to explain the PDC.

Two variables are proposed accordingly.

- The proportion of commute land use

The proportion of commute land use has been verified to have significant effect on the PDC [6], so also selected in this paper, defined as,

$$r_{i,w}=\frac{s_{i,w}}{\sum_{n}s_{i,m}}$$ (6)

where:

*r*_*i*,*w*_ is the ratio of commute land use near station *i*,

*s*_*i*,*w*_ is the area of commute land use near station *i*,

*s*_*i*,*m*_ is the area of type *m* land use near station *i*.

- Land-use function complementarity index

The mutual influence between stations is expected to alter the metro ridership in a single station, considering the fact that stations serving the same function of land use can be competitive, exclusively for adjacent stations [16]. We assume such a competition also affects the peak deviation phenomenon. Therefore, a land-use function complementarity index is introduced to describe the relationship of the land use function around a single station with that along the whole network [13,25]. Given that the proportions of commuting and non-commuting travels determine the peak deviation to a certain extent, we look at the land use function complementarity for commute land use in particular.

$$l_{i,w}=\frac{s_{i,w}/\sum_{m=1}^{M}s_{i,m}}{\sum_{i=1}^{N}s_{i,w}/\sum_{i=1}^{N}\sum_{m=1}^{M}s_{i,m}}$$ (7)

where:

*l*_*i*,*w*_ is the land-use function complementarity index for commute land use near station *i*,

*s*_*i*,*m*_ is the area of type *m* land use function around station *i*,

*M* is total number of land use function types,

*N* is total number of stations on the network.

#### Betweenness centrality

Network structure measures evaluate the efficiency of the network arrangement, which have been confirmed to show effects on the performance of transport systems [26,27]. Existing studies on transit ridership forecasting show that the passenger flows of transfer stations is larger than that of the ordinary stations, controlling the land use variables [28,29]. They normally identify the transfer stations by setting a dummy variable [30,31], which however ignores the spatial features of transfer stations on the metro network. To avoid this issue, this paper employs the concept of betweenness centrality in the graph theory as a reference, defined as,

$$BC_{i}=\underset{s\neq t}{\sum }\frac{\varepsilon_{s\underset{i}{\to }t}}{\varepsilon_{s\to t}}$$ (8)

where:

*BC*_*i*_ is the betweenness centrality of station *i*,

$\varepsilon_{s\underset{i}{\to }t}$ is the number of the shortest routes from station *s* to station *t* passing through station *i*,

*ε*_*s*→*t*_ is the number of the shortest route from station *s* to station *t*.

#### Other variables

Three other variables are also included.

- Developed or undeveloped land
  There is a previous study verified that undeveloped land around stations have strong relationship with the PDC correspondingly [6]. Thus, we build a dummy variable here to illustrate whether a station has undeveloped land nearby.
- Distance to the city center
  From the perspective of peak formation, even for commuting trips, residents in the marginal area may need to have an earlier departure time due to a longer travel time, compared to those living in town. Distance to the city center is also set as one of independent variables.
- Type of ridership
  Four types of ridership are recognized in this paper regarding the boardings and alightings in the morning and evening, that is to say boardings in the morning, alightings in the morning, boardings in the evening, and alightings in the evening. An ordinal variable is set to label these types of metro ridership.

### LSSVM method

Due to the nonlinear relationship between the metro ridership and its influencing factors [32], as one indicator of relationship characteristics, the peak deviation is difficult to be explained using a simple linear statistical model. Nonlinear methods, e.g., artificial neural network, support vector machine, and the like, can be a better choice.

Comparatively, the artificial neural network is widely used in the field of intelligent transportation systems [33,34], but still has some insurmountable defects, such as overlearning, or easily trapped into local optimization [35]. While the support vector machine (SVM) shows a strong generalization ability [36], and has been applied into traffic volume forecasting [37]. But this method runs slowly when dealing with a large sample [38]. The improved algorithm, the least-square support vector machine (LSSVM), can fit the data in segments, which allows to reduce the calculation dimensions for data fitting, and save the calculation time and increase the fitting accuracy simultaneously as a result [39,40]. The LSSVM and its improved models [41,42] have been used for traffic flow prediction [43,44], real-time traffic information extraction [45], importance evaluation of nodes in complex networks [46], regional risk prediction [47], and so on. Specially, in the field of metro system, the LSSVM has been applied to predict the time change law of passenger flows [32,36,48]. Since the peak deviation for metro stations belongs to a more detailed scope of the station-level metro ridership studies and its related work is still in the initial stage, the LSSVM method is considered to be applied in this study.

As a commonly used learning algorithm, the LSSVM model is often used to find the optimal solution in nonlinear problems. Given a data set (*x*_*i*_, *y*_*i*_), *i* = 1,2,…,*N*, *x*_*i*_∈*R*_*n*_, *y*_*i*_∈*R*, where *x*_*i*_ is the *i*^*th*^ station’s input variables. Therefore, the *x*_*i*_ is the vector containing one element for each of the independent variables, *y*_*i*_ is the *i*^*th*^ station’s output value corresponding to *x*_*i*_, which is the PDC, and N is the sample number of the station.

The LSSVM model is usually formulated as follows,

$$\underset{\omega ,b,\delta }{\min}j(\omega ,\delta )=\frac{1}{2}\omega^{T}\cdot \omega +\gamma \cdot \overset{N}{\underset{i=1}{\sum }}\delta^{2}$$ (9)

subject to:

$$y_{i}=\omega^{T}\cdot \Phi (x_{i})+b+\delta_{i}\;(i=1,2,\ldots ,N)$$ (10)

where:

Φ(*x*_*i*_) is a nonlinear conversion of independent variable *x*_*i*_ mapped to the characteristic space,

*ω* is the weight matrix,

*T* is the transpose calculation,

*γ* is the penalty parameter, which is applied to restraint the estimated error,

*δ*_*i*_ is the error,

*b* is the bias term.

We use a Lagrange function to resolve the optimization issue, described as,

$$L(\omega ,b,\delta ,\alpha )=\frac{1}{2}\omega^{T}\cdot \omega +\gamma \cdot \overset{N}{\underset{i=1}{\sum }}\delta_{i}^{2}-\overset{N}{\underset{i=1}{\sum }}\alpha_{i}\cdot [\omega^{T}\cdot \Phi (x_{i})+b+\delta_{i}-y_{i}]$$ (11)

where:

*α*_*i*_ is the Lagrange multiplier.

In order to satisfy the conditions for an optimal solution, the equations below should be satisfied,

$$\frac{\partial L}{\partial \omega }=0\Rightarrow \omega =\overset{N}{\underset{i=1}{\sum }}\alpha_{i}\cdot \Phi (x_{i})$$ (12)

$$\frac{\partial L}{\partial b}=0\Rightarrow \omega =\overset{N}{\underset{i=1}{\sum }}\alpha_{i}=0$$ (13)

$$\frac{\partial L}{\partial \delta }=0\Rightarrow \alpha_{i}=\gamma \cdot \delta_{i}$$ (14)

$$\frac{\partial L}{\partial \alpha }=0\Rightarrow \omega^{T}\cdot \Phi (x_{i})+b+\delta_{i}-y_{i}=0$$ (15)

By purging *ω*, *δ*_*i*_, *α*_*i*_ and *b* will be acquired based on the Karush-Kuhn-Tucker (KKT) terms,

$$\left[\begin{array}{cccc} 0 & 0 & \cdots & 1\\ 1 & K(x_{1},x_{1}+\frac{1}{\gamma }) & \cdots & K(x_{1},x_{N})\\ \vdots & \vdots & \ddots & \vdots \\ 1 & K(x_{N},x_{1}) & \cdots & K(x_{N},x_{N}+\frac{1}{\gamma }) \end{array}][\begin{array}{c} b\\ a_{1}\\ \vdots \\ a_{N} \end{array}]=[\begin{array}{c} 0\\ y_{1}\\ \vdots \\ y_{N} \end{array}\right]$$ (16)

where:

$K_{x_{i},x_{i}}$ is the kernel function and satisfies the Mercer circumstance, represented by,

$$K(x_{i},x)=\Phi (x_{i})\cdot \Phi (x)$$ (17)

where:

Φ(*x*_*i*_)⋅Φ(*x*) is the inner product in high-dimensional feature space.

In particular, the radial basis function (RBF) is the most effective common kernel functions in various applications, so it is applied in this study.

$$K(x_{i},x)=\exp\frac{-\|x_{i}-x{\|}^{2}}{2\sigma^{2}}$$ (18)

where:

*σ* is the breadth of the RBF.

Therefore, the LSSVM can be expressed as follows,

$$f(x)=\sum_{i=1}^{N}\alpha_{i}\cdot K(x_{i},x)+b$$ (19)

The *γ* and the *σ* are the parameters that need to be calibrated.

The continuous independent variables must be normalized before input.

$$\bar{Z_{i}}=\frac{Z_{\max}-Z_{i}}{Z_{\max}-Z_{\min}}$$ (20)

where:

*Z*_*i*_ is the original value,

$\bar{Z_{i}}$ is the normalized value of *Z*_*i*_, *Z*_*max*_ and *Z*_*min*_ is the maximum and minimum value of *Z*_*i*_, respectively.

The dummy variable identifying the undeveloped land remain unchanged.

## Case study

### Data collection

This study chooses the 2021 metro system in Xi’an, China as case, which includes 153 stations with a total length of 255.49 km. The acquired data contains,

- The geographical information of the metro stations.
  The data are extracted from Baidu Map, a widely use web mapping platform in China [49].
- Metro smart card data collected from Automatic Fare Collection (AFC) System
  Metro smart card data during the workdays from May 10 to 31 in 2021 (16 days in total) are acquired from Xi’an Rail Transit Group Company Limited, which includes attributes like card ID, boarding/alighting time slot, boarding/alighting station number, and so on. The temporal distribution of each station can be calculated based on the aggregations grouped by the boarding/alighting time slot specific to the station numbers.
- The Point of Interest (POI) data
  The POI data is also extracted from Baidu Maps [50], which is used to identify the type of land use function for different buildings in the catchment area of the metro stations (set as 800 meters in this study [51,52]. Ten types of land use functions are counted, including residential, commercial, enterprise, recreational, transport, medical, parks, education, industrial, and agricultural, based on the official classification criteria of the POI data. The area of each type is then measured based on the building contour line imported from Baidu maps as well as the plot ratio defined by the corresponding national construction standards.

### Statistical descriptions of data

#### Dependent variables

The city peak of the metro network in Xi’an during the morning and evening is 7:30–8:30 a.m. and 18:00–19:00 p.m., respectively, see Fig 1. We count the number of boardings and alightings for each station during these two periods and calculate the average hourly ridership, as $P_{i,t^{c}}$ represents in Eq 5.

The maximum hourly boardings and alightings in the morning and evening are also aggregated for each station, taking 12:00 pm as the split line, which gives the ridership during the station peak, represented by $P_{i,t_{i}^{s}}$ as shown in Eq 5.

**Fig 2:**
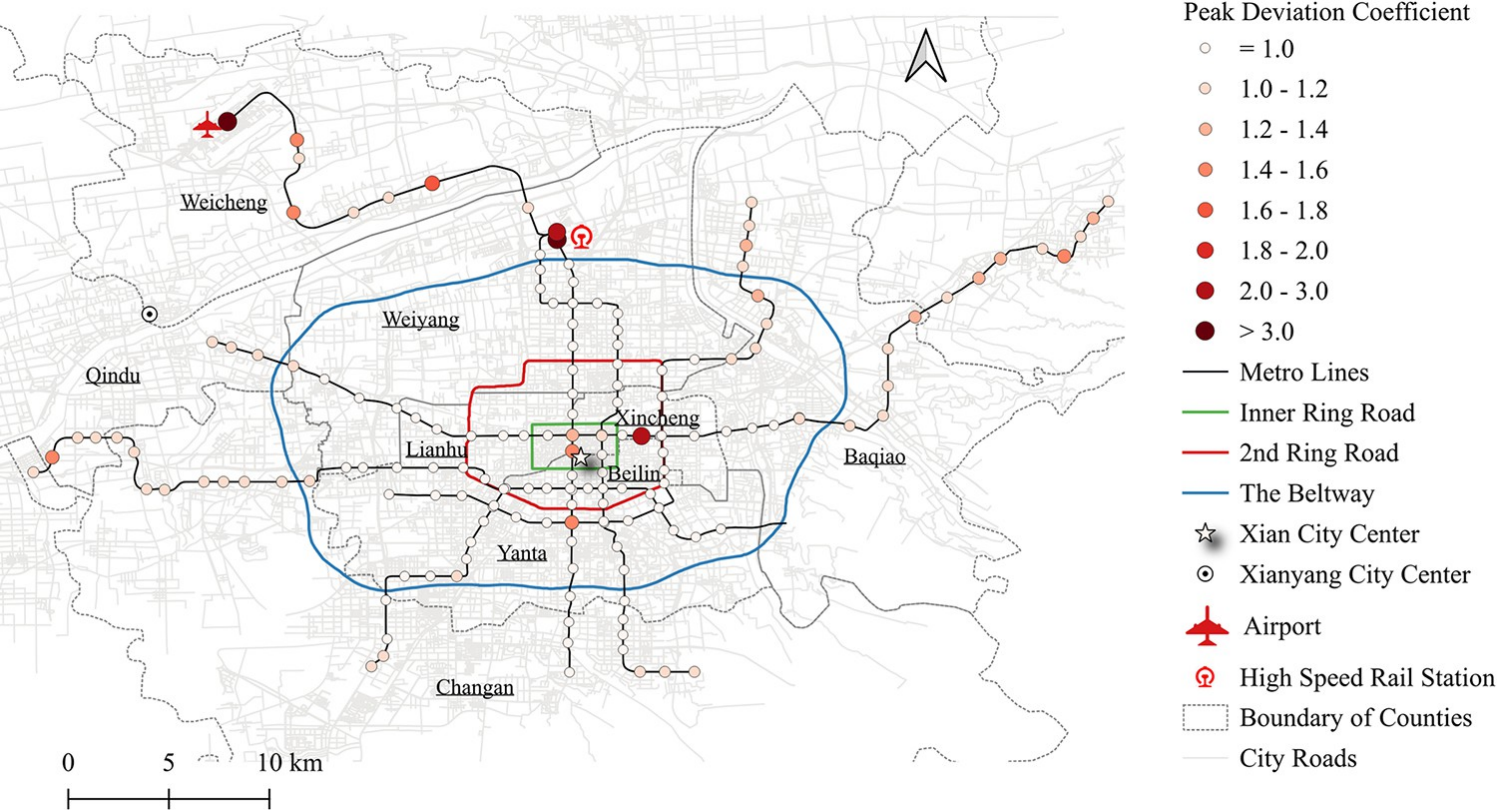
An illustration of the study area with a visualization of the peak deviation coefficient (PDC) regarding morning boardings.

**Fig 3:**
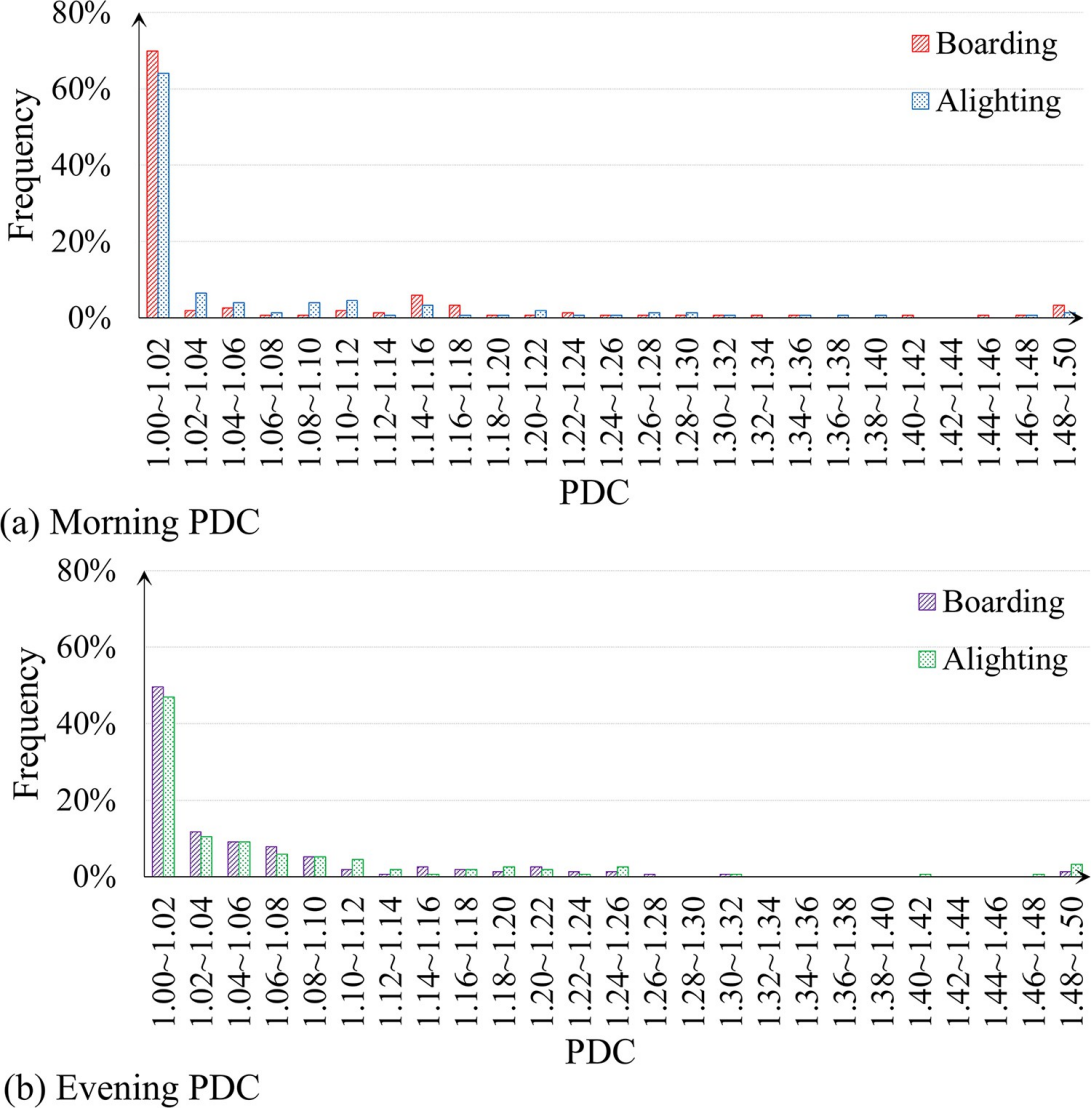
Histograms of peak deviation coefficient (PDC) values for metro stations regarding boardings and alightings in the morning and evening, respectively.

The PDC can be then calculated, specific to the boardings and alightings in the morning and evening, respectively. The spatial distribution of the PDC for metro station boardings during the morning is shown in Fig 2. Its histogram is also displayed in Fig 3 in addition to that of the other three types of PDCs. It is apparent that most of stations barely have a significant peak deviation, for instance, about 70% of the stations hold a PDC less than 1.02 for the morning boardings. Exceptions do exist, in particular for the stations near the external transportation hubs, e.g., the high-speed rail station or the airport, their PDCs are greater than 1.48. Comparatively, the peak deviation issue in the evening is more severe than the morning that around 53% of the stations have their PDCs greater than 1.02. More details are discussed in the Section Case study.

#### Independent variables

**Fig 4:**
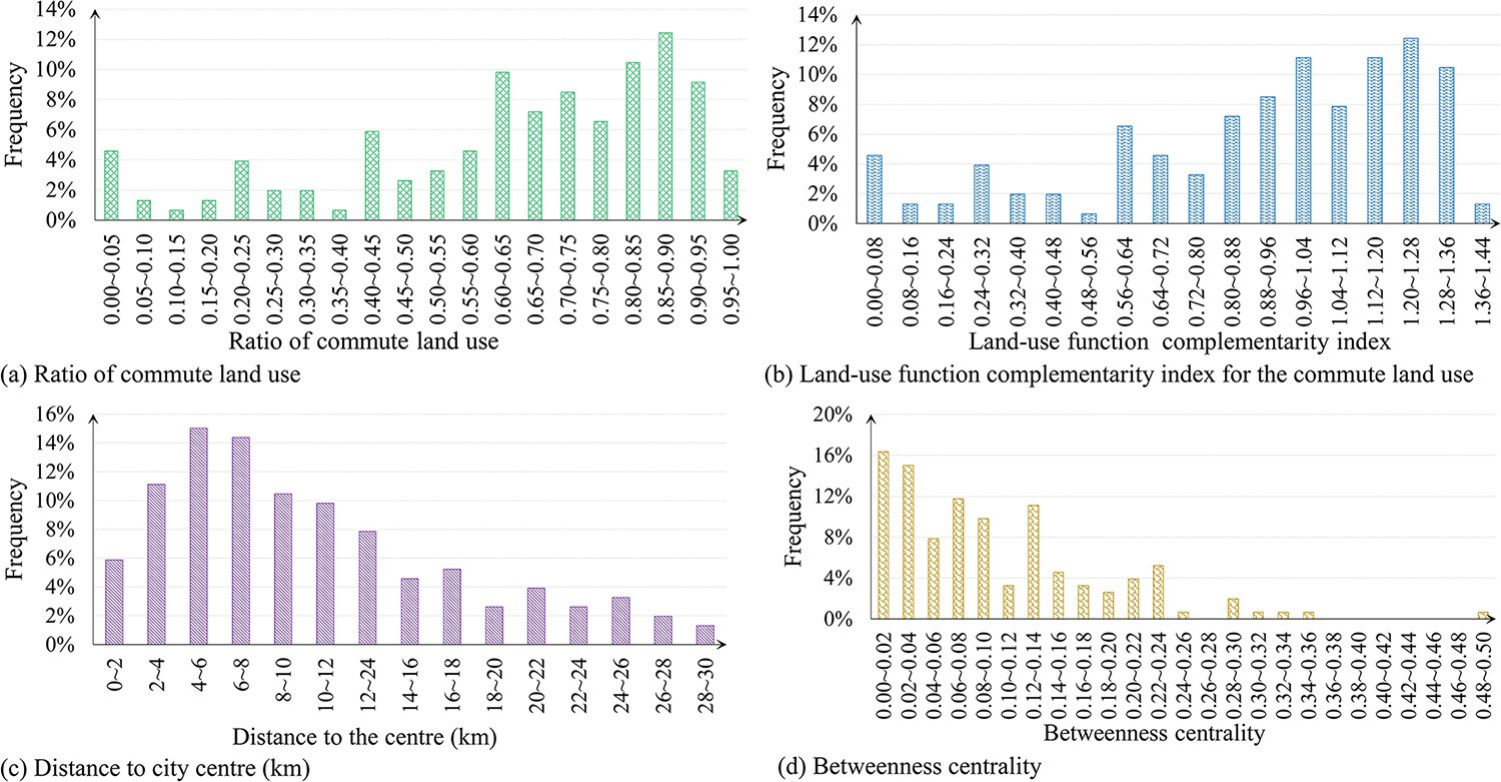
Histograms of the selected independent variables.

Six independent variables are selected in this study. Their definitions as well as statistical descriptions are summarized in Table 1. Fig 4 shows the histograms of the continuous variables.

**Table 1:** Statistical description of the independent variables.

Independent variables	Description	Min	Max	Mean
Ratio of commute land use	The area of commute land use over the total area of all types of land use in the catchment area of a station (Eq 6).	0.000	0.980	0.635
Land-use function complementarity index	The ratio of commute land use of a single station to that of the network (Eq 7).	0.000	1.397	0.905
Betweenness centrality	The number of the shortest paths passing through a station over the total number of the shortest path (Eq 8).	0.000	0.485	0.099
Developed or undeveloped land	A dummy variable, 0: no undeveloped land in the catchment area of a station; 1: otherwise.	0	1	-
Distance to city center	The Euclidean distance from each station to the Bell Tower, the landmark building of Xi’an city center.	0.152	29.885	10.413
Type of ridership	An ordinary variable: 1: number of boardings in morning; 2: number of alightings in morning; 3: number of boardings in evening; 4: number of alightings in evening.	1	4	-

Note also that the ratio of the commute land use and the land-use function complementarity index are defined all based on the area of the commute land use, so cannot be applied simultaneously in the same model due to multicollinearity issues. See Fig 4A and 4B that similar trends can be observed. For this reason, two models are built using each of the commute land use variables, in addition to other variables. To be more specific,

- Model 1 includes the ratio of the commute land use.
- Model 2 includes the land-use function complementarity index.

Our hypothesis is that Model 2 should perform better, as it further integrates the competition among the commute land use along the network when explaining the PDC.

## Results

As four types of PDCs are recognized, namely morning boardings, morning alightings, evening boardings, and evening alightings, 153 stations are rearranged correspondingly, which obtains 153×4 rows of data. 70% of the data are randomly selected as the training set, and the rest are used as the test set.

**Fig 5:**
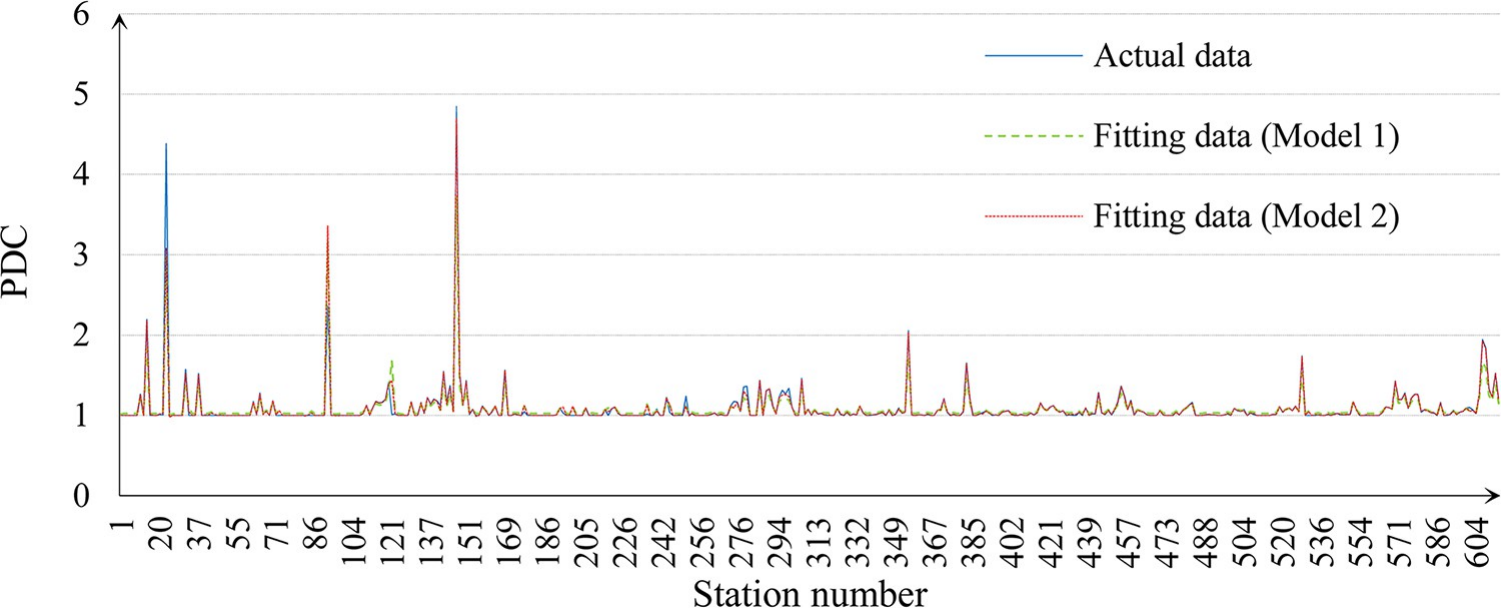
Results of the training set.

**Fig 6:**
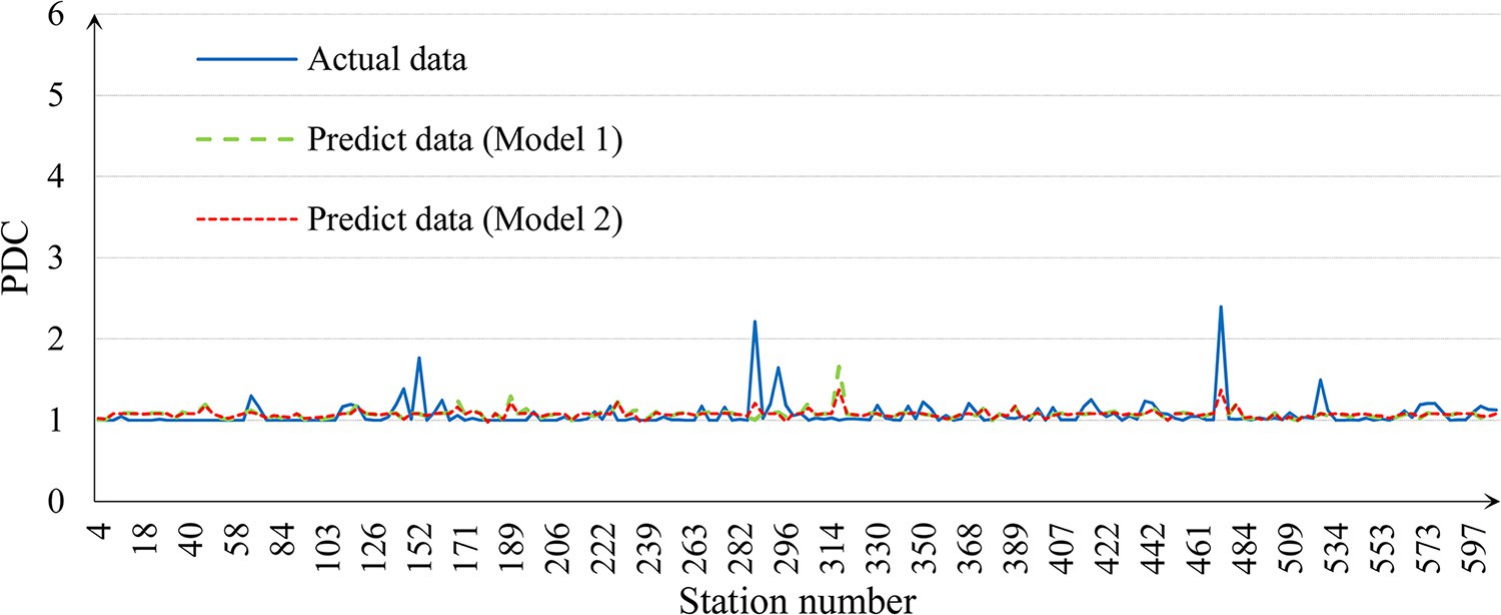
Results of the test set.

The Matlab LSSVM toolbox is used to calibrate the parameters for both Model 1 and Model 2 using the training set. The results are summarized in Table 2 and Fig 5. From the results, it clearly shows that Model 2 explains the PDC much better, compared to Model 1, that improves the Adj. R^2^ by 17.1% (changing from 0.777 to 0.910) and reduces the root mean square error by 25.6% (changing from 11.34% to 8.43%). The predicted results accordingly based on the test set are shown in Table 3 and Fig 6 which further verify the better performance of Model 2 that its relative error is much lower than Model 1.

**Table 2:** Fitting results of the training set.

	*b*	*γ*	*σ* ^2^	Adj. *R*^2^	Root mean square error
**Model 1**	-0.034	2.075	0.002	0.777	11.34%
**Model 2**	-0.018	50.492	0.009	0.910	8.43%

**Table 3:** Predicted results of the test set.

	Maximum relative error	Minimum relative error	Average relative error	Root mean square error
**Model 1**	65.49%	-56.14%	1.59%	18.00%
**Model 2**	37.27%	-45.58%	1.54%	15.52%

These support our hypothesis that considering the inter-station correlations helps to better understand and predict the formation of the PDC.

### Sensitivity analysis

**Fig 7:**
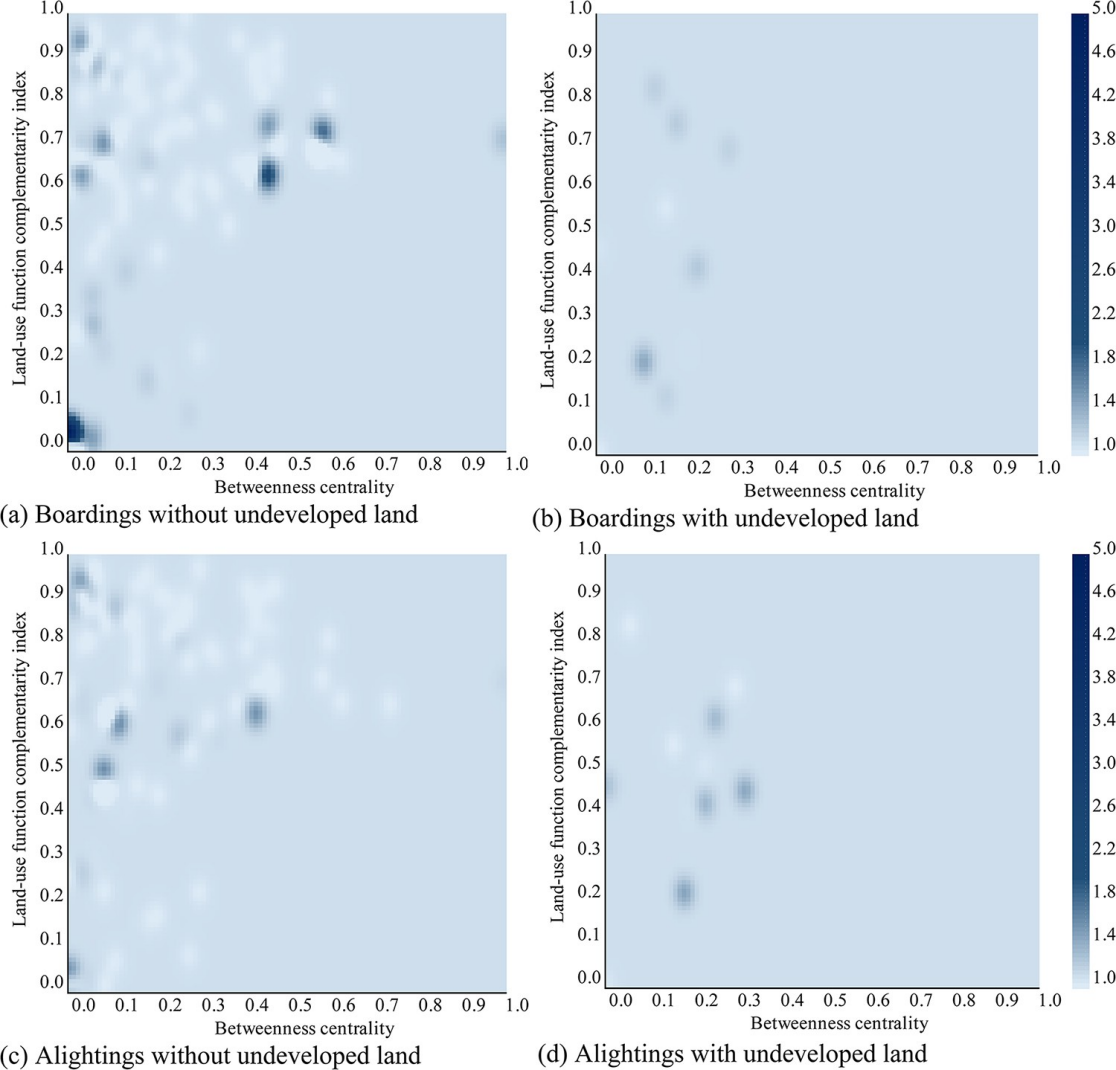
Influence of land-use function complementarity index and betweenness centrality on the PDC in the morning.

**Fig 8:**
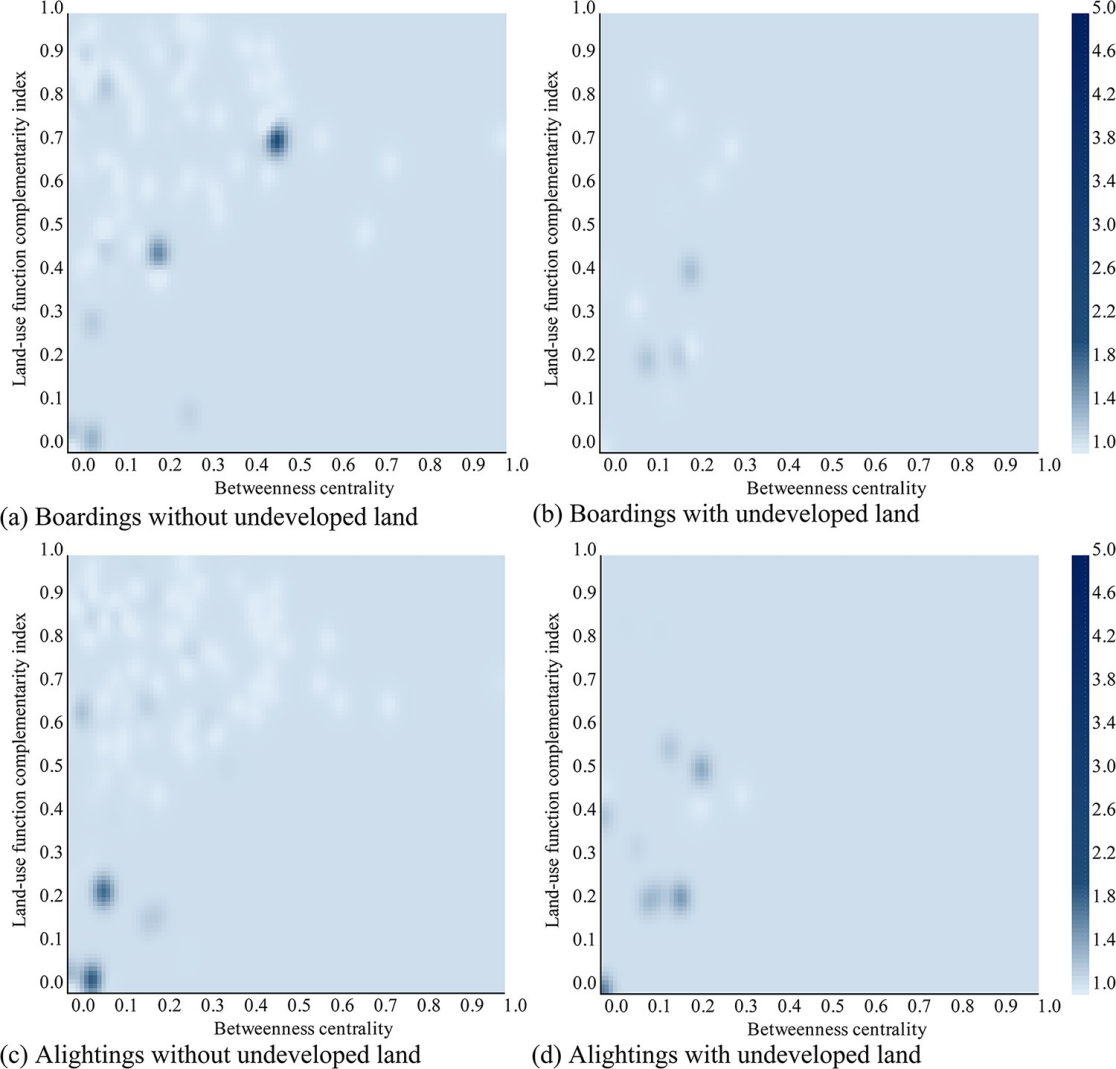
Influence of land-use function complementarity index and betweenness centrality on the PDC in the evening.

Based on the estimates of Model 2, a sensitivity analysis is conducted to explore the effects of different independent variables on the PDC. Controlling the dummy variable (developed or undeveloped land) as well as the ordinary variable (the type of ridership) in the model, the PDC estimates concerning the changes of betweenness centrality and land use function complementarity index are visualized in Figs 7 and 8 for the morning and evening scenarios, respectively. As the continuous independent variables are required to be normalized before input in the LVSSM model, so both the x- and y- axis are all ranged from 0.0 to 1.0.

As we can see, from either the vertical or the horizontal point of view, the color on all plots does not show gradual changes, demonstrating the nonlinear effects of betweeness centrality and land use function complementarity index on the PDC.

Specifically, in most cases, the PDC values are between 1.0 and 1.4, which tells that the station peak nearly overlaps with the city peak. This is inevitable, because the city peak is the time period that travel activities are most likely to happen for the whole network. But noticeable high-PDC clusterings cannot be ignored. For instance, in Fig 7A, the maximum PDC value emerges when the land-use function complementarity index less than 0.1 and the betweenness centrality less than 0.05, which indicates that stations with small areas of commute land use near the end of the metro lines should have a more severe peak deviation of passenger boardings in the morning. This recalls the stations close to the airport or the high-speed rail station, consistent with the finding from the statistical description of the dependent variable, that schedules of flights or high-speed trains matter more than the commute travel activities city-wide. The same high-high cluster can be found for the alightings in the evening, see Fig 8C. Also, a higher value of PDC also appears when the land-use function complementarity index is in the range of 0.6 and 0.7 and the betweenness centrality is between 0.4 and 0.5. This refers to the case of the suburban region where commute land use occupies, so generates commute trips. But considering the longer travel time from the suburbs to e.g., the city center, the peak deviation arises to catch up the working hours. The evening boarding shares a similar high-high cluster location, see Fig 8A.

The variations of PDC for stations without undeveloped land is more drastic than those with undeveloped land along the changes of the betweenness centrality and the land use function complementarity index. It is not surprising as the undeveloped land is mainly located in the marginal area of Xi’an city and the stations there have much bounded capabilities to generate or attract commute trips. So even peak deviation issue occurs in those stations, the severity of it can be limited. It is worth to mention that no apparent changes can be observed for the ’with undeveloped land’ scenarios, either boardings or alightings in the morning or the evening, when the betweenness centrality is greater than 0.5. A reasonable explanation is that there are no stations inside of the 2nd-ring road having undeveloped land nearby. Thus, it lacks of data to interpret such a condition.

## Conclusion

This study concentrates on the deviation of peak hours in metro stations comparing with the city peak, and investigates what are the influencing factors and how the influencing factors affect the peak deviation. To do so, a peak deviation coefficient is proposed to quantitatively describe the severity of the peak deviation phenomenon. The LSSVM method is applied considering the nonlinear relationship between the metro ridership and its determinants.

Overall, the LSSVM is verified to be an effective method when explaining the PDC for metro stations, as, for the two proposed models, the adjusted *R*^2^s are higher than 0.7 with a root mean square error less than 12%. The predictions accordingly also show a quite satisfied accuracy.

Commute travels are considered as the main cause of peak deviation. Two variables are defined based on the area of land use regarding commute travels in the catchment area of metro stations, the ratio of commute land use and the index of commute land-use function complementarity, the latter of which is newly proposed in this study that describes the relationship of the commute land use around an individual station with that along the whole network. The results illustrate that the land-use function complementarity index can better explain and predict the severity of peak deviation phenomenon, controlling other independent variables in the model. This indicates that inter-station correlations should be concerned when interpreting the station-level ridership or its corresponding temporal trends. Future research can further explore if the competitive and complementary relationship between stations affects the spatial distribution of urban rail transit demand.

The sensitivity analysis accordingly is conducted to examine the variation of the predicted PDC with the changes of the selected independent variables, and non-linear effects are demonstrated. From the details, the station peak matches the city peak for most of the stations. But there are exceptions, particularly for the ones with no undeveloped land nearby, that the clustering of high PDC values shows up in some of the ’low betweenness central & low land-use function complementarity index’ or ’medium betweenness central & medium land-use function complementarity index’ composites of settings, which brings challenges to the traditional basis of station design.

Based on the requirement of the *Transit Capacity and Quality of Service Manual*, the size of subway stations should be decided according to the predicted maximum ridership at a given time. The *Code of Metro Ridership Forecast* in China also stipulates that the capacity of the internal facilities in metro stations should consider the extra peak hour factor (EPHF), which equals to 4 times of the passenger flow during the busiest 15 min of the city peak over the total passenger flow during the peak hour [7]. The question here is if the EPHF can be employed solely for station design regarding the size and facility capacity regardless the effects of the PDC. From the definitions, the PDC depicts the disparity on the horizontal axis comparing the city peak with the station peak, while the EPHF expresses the peak sharpness on the vertical axis. Quantitatively speaking, stations that have a higher ratio of commercial or recreational land might have a higher PDC due to the lack of commute travels but hold a lower level of EPHF considering the randomness of passenger flows [53]. This emphasizes the necessity to explicit their integrated application in subway station planning and design. A simple way is to first use the PDC to shift the city peak ridership to the station peak, and then adopt the EPHF based on the station peak ridership to increase the risk resistance of the station. Future research might come up a more thorough method based on a series of comprehensive analysis and result comparison.

In addition, we would also like to extend this study to further consider the effects of peak deviation issues on the station-to-station metro ridership, given the fact that the studies of OD-based metro ridership are rare [54] and OD ridership shows more complex regularity comparatively, which helps to understand the intrinsic connections of metro stations.

## Supporting information

S1 Dataset (XLSX)
